# Effects of a Caffeine-Containing Energy Drink on Simulated Soccer Performance

**DOI:** 10.1371/journal.pone.0031380

**Published:** 2012-02-14

**Authors:** Juan Del Coso, Víctor E. Muñoz-Fernández, Gloria Muñoz, Valentín E. Fernández-Elías, Juan F. Ortega, Nassim Hamouti, José C. Barbero, Jesús Muñoz-Guerra

**Affiliations:** 1 Camilo José Cela University, Exercise Physiology Laboratory, Madrid, Spain; 2 University of Castilla-La Mancha, Exercise Training Laboratory, Toledo, Spain; 3 Spanish Anti-doping Agency, Doping Control Laboratory, Madrid, Spain; 4 University of Castilla-La Mancha, Exercise Physiology Laboratory, Toledo, Spain; 5 University of Granada, Campus of Melilla, Melilla, Spain; Universidad Europea de Madrid, Spain

## Abstract

**Background:**

To investigate the effects of a caffeine-containing energy drink on soccer performance during a simulated game. A second purpose was to assess the post-exercise urine caffeine concentration derived from the energy drink intake.

**Methodology/Principal Findings:**

Nineteen semiprofessional soccer players ingested 630±52 mL of a commercially available energy drink (sugar-free Red Bull®) to provide 3 mg of caffeine per kg of body mass, or a decaffeinated control drink (0 mg/kg). After sixty minutes they performed a 15-s maximal jump test, a repeated sprint test (7×30 m; 30 s of active recovery) and played a simulated soccer game. Individual running distance and speed during the game were measured using global positioning satellite (GPS) devices. In comparison to the control drink, the ingestion of the energy drink increased mean jump height in the jump test (34.7±4.7 *v* 35.8±5.5 cm; *P*<0.05), mean running speed during the sprint test (25.6±2.1 *v* 26.3±1.8 km · h^−1^; *P*<0.05) and total distance covered at a speed higher than 13 km · h^−1^ during the game (1205±289 *v* 1436±326 m; *P*<0.05). In addition, the energy drink increased the number of sprints during the whole game (30±10 *v* 24±8; *P*<0.05). Post-exercise urine caffeine concentration was higher after the energy drink than after the control drink (4.1±1.0 *v* 0.1±0.1 µg · mL^−1^; *P*<0.05).

**Conclusions/significance:**

A caffeine-containing energy drink in a dose equivalent to 3 mg/kg increased the ability to repeatedly sprint and the distance covered at high intensity during a simulated soccer game. In addition, the caffeinated energy drink increased jump height which may represent a meaningful improvement for headers or when players are competing for a ball.

## Introduction

Caffeine (1,3,7-trimethylxanthine) is one of the most consumed drugs in sports in our days. A recent study has shown that 3 out of 4 elite athletes consume caffeine prior to competing, based on the post-exercise urinary caffeine concentrations of 20,686 urine samples obtained for doping analysis [1]. However, the manner in which athletes consume caffeine is diverse. Caffeine is present in coffee and chocolate beans, tea leaves and kola nuts and so can be consumed from natural sources (coffee, tea, chocolate, etc). In addition, caffeine can be artificially synthesized and included in food and drinks, like the recently created energy drinks. These beverages contain moderate amounts of caffeine (32 mg · 100 mL^−1^) in addition to carbohydrates, taurine, glucoronolactone and B- group vitamins [2]. Due to their low cost, accessibility, and the relatively low frequency of deleterious side-effects derived from their consumption, caffeine-containing energy drinks have become the most popular supplement in the sports population, with a prevalence of 73% in American college athletes [3], 75% in Canadian Varsity athletes [4] and 42% in British elite athletes [5].

Whereas the outcomes of caffeine ingestion (from natural sources and pills) are well known [6], [7], [8], [9], the effects of caffeine-containing energy drinks on sports performance have been the object of fewer studies. The first report concerning the effects of energy drinks on physical performance was carried out by Alford and co-workers in 2001 [10]. These authors found that ∼1 mg of caffeine per kg of body weight (one 250-mL serving of an energy drink) improved reaction time, alertness and aerobic and anaerobic performance. In contrast, subsequent investigations using energy drinks have shown that ∼1 mg/kg of caffeine is not enough to enhance maximal oxygen uptake [11], peak power during three repetitions of the Wingate test [12], [13] or running velocity during 24 “all-out” sprints [14]. In addition, the ingestion of ∼2 mg/kg of caffeine in the form of an energy drink was ergogenic during a cycling time trial [15] but did not prolong time-to-exhaustion during a running test at 80% VO_2max_ [16].

The ergogenic effect of caffeine on endurance activities has been typically demonstrated with doses from 3 to 9 mg/kg [17], while the ingestion of 1 mg/kg of caffeine did not improve performance [18]. Similar results have been found in team-sports specific activities: the ingestion of 6 mg/kg of caffeine increased repeated sprint ability [19], [20] while the ingestion of 1 mg/kg of caffeine did not alter repeated sprint performance [14]. Since the ingestion of one serving of an energy drink (typically 250-mL that contains 80 mg of caffeine) provides a dose of ∼1 mg/kg of caffeine in a man of average weight, previous results about the inefficacy of energy drinks to improve performance [11], [12], [13], [14], [16] may be explained by the low dose of caffeine provided for the subjects.

During a soccer match, players combine periods of high-intensity exercise interspersed with periods of lower-intensity exercise or recovery. Thus, the ability to perform repeated sprints with minimal recovery between sprint bouts is one of the most crucial capacities for team sport athletes [21]. In addition, an enhanced ability to repeat sprints is related with playing at a higher competitive level, especially in soccer [22]. The aim of the present study was to investigate the effects of a caffeine-containing energy drink (3 mg/kg of caffeine) on the capacity of soccer players to repeat sprints. This physical ability was tested during a soccer-specific test and during a simulated soccer match. We hypothesized that 3 mg of caffeine per kg of body weight in the form of an energy drink would increase the capacity of soccer players to perform repeated sprints.

## Methods

### Subjects

Nineteen semiprofessional soccer players from the same team volunteered to participate in the study. They had a mean ± SD age of 21±2 yrs, body mass of 67±2 kg, height of 173±6 cm and maximal heart rate of 197±12 bpm. All participants had previous soccer experience of at least 5 yrs and had trained for ∼2 h · day^−1^, 4–5 days · week^−1^ (including a weekly competition) during the previous year. No participant had a previous history of cardiopulmonary diseases or was taking medications during the study. Participants were non-smokers but all of them were light caffeine consumers (<60 mg per day, ∼1 cup of coffee). Initially, 22 players were recruited (to complete the simulated game), but one soccer player did not finish the protocol and the data from the two goalkeepers were not included in the analysis due to the difference in their movement with the outfield players.

### Ethics Statement

Participants were fully informed of any risks and discomforts associated with the experiments before giving their informed written consent to participate. The study was approved by the Virgen de la Salud Hospital Research Ethics Committee, Toledo (Spain) in accordance with the latest version of the Declaration of Helsinki.

### Experimental Design

Each player performed 2 experimental trials under the same experimental conditions (24°C WBGT; 27°C dry temperature; 50% relative humidity). On one occasion, participants ingested 630±52 mL of a caffeine-containing energy drink (sugar-free Red Bull®; 32 mg of caffeine per 100 mL). The volume of the energy drink was individually set to provide a dose of 3 mg of caffeine per kg of body mass. On another occasion, players ingested the same volume of a control drink (sugar-free decaffeinated Pepsi®) with no caffeine content (0 mg/kg). The beverages were ingested 60-min before the onset of the experimental trials to allow complete caffeine absorption and they were provided in opaque plastic bottles to avoid identification. However, the taste of the drinks was slightly different and there still remains the possibility that the players were able to identify the drinks. To avoid the “placebo effect” in the experimental trials, we did not inform the subjects about the names (i.e. trade marks) of the drinks and we presented both of them as having similar ergogenic properties. The participants were divided into two groups and the order of the experimental trials was counterbalanced and randomized. Thus, players of the same soccer team received different experimental treatments. Trials were separated by one week to allow for a complete recovery. An alphanumeric code was assigned to each trial to blind participants and investigators to the drink tested. This code was unveiled after the analysis of the variables.

### Experimental Protocol

The day before each experimental trial, participants refrained from strenuous exercise and adopted a similar diet and fluid intake regimen. Participants were encouraged to refrain from all dietary sources of caffeine (coffee, cola drinks, chocolate, etc) and alcohol for 48 hours before testing. Verbal reminders were given to ensure compliance. In addition, participants were instructed to have their habitual breakfast at least two hours before the start of the experimental trials. At 10.00 AM, participants arrived at their habitual training stadium and voided in a sterilized container. A representative urine sample was obtained and frozen at −80°C for future analysis. After that, participants were nude-weighed (Sense, Tefal, France) and the beverage assigned for the trial was individually provided and consumed. Then, players dressed in a T-shirt, shorts, soccer socks and cleats. They also wore a GPS/Accelerometer/HR device inserted in a purpose built back-pack (GPS, SPI PRO X, GPSports, Australia) and a heart rate belt (Polar®, Finland) attached to their chest. Participants then performed a standardized warm-up and began the performance tests (60 min after beverage ingestion). The performance tests included maximal jump height, maximal running speed and a simulated game (see below). The participants were familiarized with the performance test used in this investigation and had worn the GPS device in previous official and friendly games. Post-exercise nude body weight was obtained within 10 min of the end of the game. Thirty minutes after the end of the game, participants voided again and a urine sample was obtained for urine caffeine concentration analysis. During each experimental trial, players drank water *ad libitum*. Players were instructed to drink only from their own individually labeled bottles and not to spit out or spill any fluid. Fluid intake was measured from the change in bottle weight using a scale (Delicia, Tefal, France). Sweat rate was estimated from body mass loss, total fluid intake and experimental trials duration.

### Fifteen seconds maximal jump test

Maximal vertical jump height was determined during a 15-s rebound jump series by measuring the flight time (0.001 s) with an infrared laser system (Optojump Next, Optojump, Spain). For this measurement, participants began in an upright position with their weight evenly distributed over both feet and their arms freely positioned to each side of the body. After a voice signal, the participants jumped vertically as high as possible and landed with both feet at the same time, repeating this jumping action for 15-s. Participants were instructed to “jump as high as possible during 15-seconds simulating header shots”. Muscle power output during the jump series was calculated using body weight, jump height and number of jumps according to the following formula [23]:
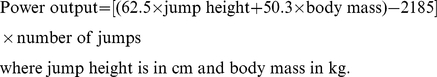



### Repeated sprint ability test

Five minutes after the end of the jump test, players performed 7×30-m running sprints with 30-s of active recovery between repetitions. Verbal feedback was given to encourage the players to produce maximal running speed in each sprint and to inform them of the recovery time remaining between sprints. Sprint performance was measured by means of a GPS device attached to the subject's back. The GPS was set to assess distance at a frequency of 15 Hz. The validity and reliability of the GPS to assess maximal running speed and repeated sprint ability has been previously verified [21].

### Simulated soccer game

Participants completed a 2×40 min simulated soccer game (without extra time at the end of each half), including a 15-min half time. The game was played on a regular artificial turf soccer field (100×65 m) with 11 players per side, while a referee took decisions on play disputes during the game. The game followed the rules of the Federation Internationale de Football Association (FIFA). Participants were divided into two soccer teams according to their outfield position to allow them to play in their habitual position. In each team, an equal number of players received the energy drink or placebo. During the game, the GPS device and heart rate belt monitored data on running distance, running speed, maximal and mean heart rate. Validity and reliability of the GPS system has been reported to apply in team sports [24]. Analyses of soccer player movement during the game were categorized as follows, based on a previous study by Castagna et al. [25]: Standing (ST: 0–0.4 km · h^−1^); walking (W: 0.5–3.0 km · h^−1^); jogging (J: 3.1–8.0 km · h^−1^); medium-intensity running (MIR: 8.1–13.0 km · h^−1^); high-intensity running (HIR: 13.1–18.0 km · h^−1^); and sprinting (SP: running speed higher than 18.0 km · h^−1^). Each displacement with a running speed higher than 18 km · h^−1^ was considered a sprint bout. The number of sprint bouts during each experimental trial was used for analysis. All the data analyses were performed with a specific software package (Team AMS software V R1.2011.6, GPSports).

### Urine analysis

After urine homogenization, a representative (3 mL) specimen of each sample was obtained and stored at −80°C. At a later date, urine was analyzed for caffeine, paraxanthine, theobromine and theophylline concentrations using an Agilent Technologies HPLC 1200 system (Santa Clara, CA, US) coupled to a triple quadrupole/ion trap mass spectrometer (MS; API 400, Q TRAP, US). Caffeine, paraxanthine, theobromine, theophylline and the internal standard (7-β-hydroxy-ethyltheophylline) were purchased from Sigma-Aldrich (Spain). For this measurement, 20 µL of the internal standard (60 µg · mL^−1^) were added to 200 µL of urine. A volume of 150 µL of mobile phase (isopropanol-acetic acid 0.1%; 3.5∶96.5, v/v) was added to the urine sample and then it was centrifuged at 3500 rpm for 10 min. After centrifugation, 10 µL of the supernatant were then directly applied to the HPLC-MS system. To calibrate the system, aqueous solutions of caffeine (ranging from 0.25 to 12 µg · mL^−1^) and paraxanthine, theobromine and theophylline (from 0.5 to 30 µg · mL^−1^) were used before each batch of samples. The lower limit for the accurate quantization of these methylxanthines was 0.25 µg · mL^−1^.

### Statistical Analysis

Data from the 15-s maximal jump test and the repeated sprint ability test were analyzed using a two-way ANOVA (beverage×repetition) with repeated measures. After a significant *F* test (Geisser-Greenhouse correction for the assumption of sphericity), differences between means were identified using Tukey's HSD *post hoc* procedure. Total running distance during the simulated game, the running distance at different speeds and the number of sprints were examined by using paired t-tests. Data on sweat rate and the urinary concentrations of caffeine, paraxanthine, theophylline and theobromine were also examined by using paired t-tests. For each difference found in this study, we have calculated the effect size (ES) proposed by Cohen. The data were analyzed with the statistical package SPSS v 18.0 (SPSS Inc., Chicago, IL). The significance level was set at *P*<0.05. The results are presented as means ± SD.

## Results

### Vertical jump height and muscle power

**Figure 1:**
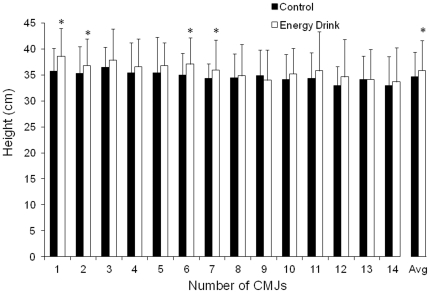
Vertical jump height during a 15-s maximal jump test. Vertical jump height during a 15-s maximal jump test with the ingestion of a caffeinated energy drink (3 mg of caffeine/kg of body weight) or the ingestion of a decaffeinated control drink. Data are mean ± SD for 19 soccer players. * Different from control (*P*<0.05).

The ingestion of the caffeinated energy drink increased mean jump height during the 15-s maximal jump test in comparison to the ingestion of the decaffeinated control drink (Figure 1; *P*<0.05). On average, soccer players' jump height was 35.8±5.5 cm with the energy drink and 34.7±4.7 with the control drink (*P*<0.05; ES = 0.23). Although there was a main effect for repetitions during the jump test (*P*<0.05), there were no interactions between beverages and repetitions (*P*>0.05). The total power generated during the 15-s jump test was higher with the energy drink than with the control drink (61.8±7.3 *v* 59.5±6.9 kW, *P*<0.05; ES = 0.33).

### Repeated sprint ability test

**Figure 2:**
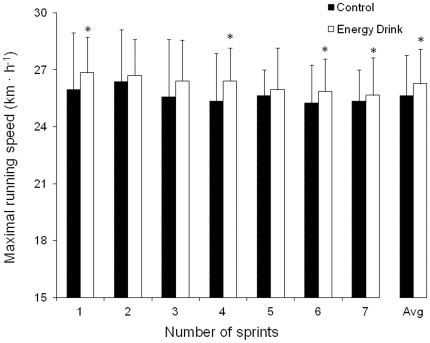
Maximal running speed during a 7×30 m sprint test. Maximal running speed during a 7×30 m sprint test with the ingestion of a caffeinated energy drink (3 mg of caffeine/kg of body weight) or the ingestion of a decaffeinated control drink. Data are mean ± SD for 19 soccer players. * Different from control (*P*<0.05).

The intake of the energy drink increased mean peak running speed during a 7×30 m sprint test (Figure 2; *P*<0.05). The average peak running speed was 26.3±1.8 km · h^−1^ with the energy drink and 25.6±2.1 km · h^−1^ with the placebo drink (*P*<0.05; ES = 0.33). There was a main effect for repetitions during the repeated sprint test (*P*<0.05) but there were no interactions between beverages and repetitions (*P*>0.05).

### Distance covered and running speed during a simulated soccer game

**Figure 3:**
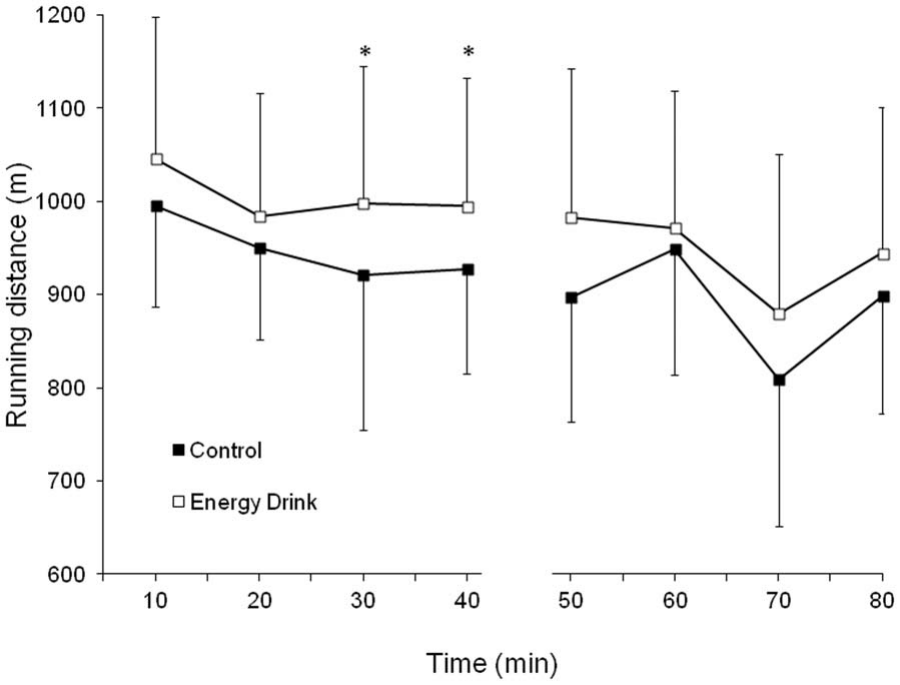
Running distance at 10 min intervals during a simulated soccer game. Running distance at 10 min intervals during a simulated soccer game with the ingestion of a caffeinated energy drink (3 mg of caffeine/kg of body weight) or the ingestion of a decaffeinated control drink. Data are mean ± SD for 19 soccer players. * Different from control (*P*<0.05).

**Figure 4:**
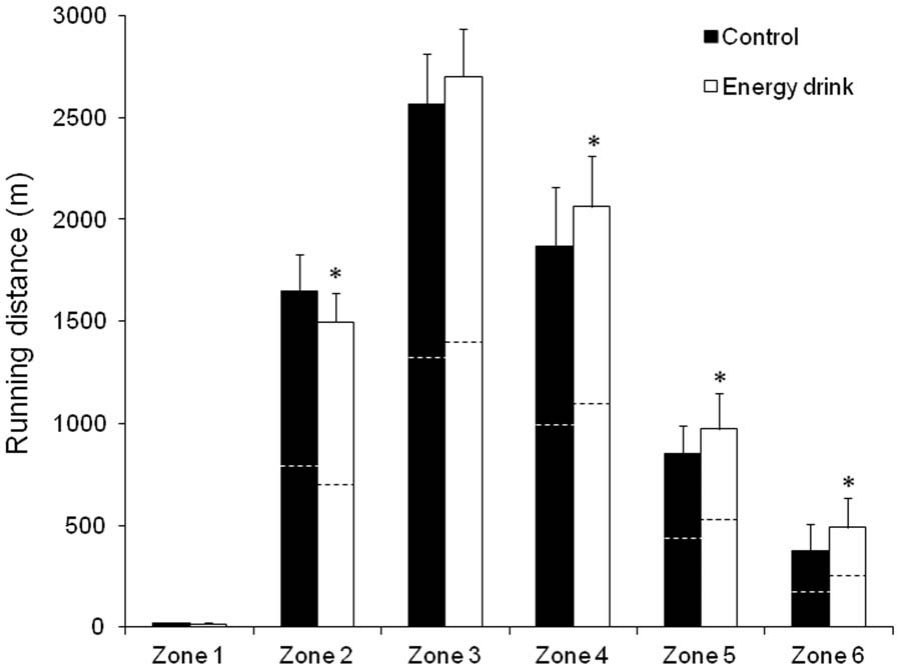
Running distance covered at different speeds during a simulated soccer game. Running distance covered at different speeds during a simulated soccer game with the ingestion of a caffeinated energy drink (3 mg of caffeine/kg of body weight) or the ingestion of a decaffeinated control drink. Data are mean ± SD for 19 soccer players. * Different from control (*P*<0.05). Dashed lines indicate the half time. Zone 1 (Standing) = 0–0.4 Km · H^−1^; Zone 2 (Walking) = 0.5–3.0 Km · H^−1^; Zone 3 (Low-Intensity Running) = 3.1–8.0 Km · H^−1^; Zone 4 (Medium-Intensity Running) = 8.1–13.0 Km · H^−1^; Zone 5 (High-Intensity Running) = 13.1–18.0 Km · H^−1^; Zone 6 (Sprinting) = Speed Higher Than 18.0 Km · H^−1^.

The total distance covered during a 2×40 min simulated soccer game was 7782±878 m after the ingestion of the energy drink and 7352±881 m after the ingestion of the control drink (*P*<0.05; ES = 0.48). Figure 3 depicts the distance covered at 10 min intervals during the whole game. The distance tended to be higher with the ingestion of the energy drink, and it was significantly higher at the end of the first half (*P*<0.05). Figure 4 illustrates the distance covered at different speeds, ranging from standing (zone 1) to sprinting (zone 6). In comparison to the controls, the utilization of the caffeinated energy drink produced a significant rise in distance covered at medium intensity running (zone 4; *P*<0.05; ES = 0.35), at high intensity running (zone 5; *P*<0.05; ES = 0.42) and sprinting (zone 6; *P*<0.05; ES = 0.51). In contrast, the energy drink reduced the distance covered by walking (zone 2; *P*<0.05; ES = −0.54) when compared to the control drink. Finally, the number of sprint bouts during the game was increased with the ingestion of the caffeine drink (30±10 *v* 24±8; *P*<0.05; ES = 0.75).

### Exercise heart rate, sweat rate and urine caffeine excretion

Maximal (196±7 *v* 197±12 bpm for energy drink and control, respectively) and averaged heart rate during the game (160±10 *v* 161±12 bpm) were unaffected by the ingestion of the energy drink. Similarly, sweat rate (1.1±0.3 *v* 1.0±0.3 L · h^−1^) was similar with the ingestion of the two experimental beverages. The ingestion of the caffeine-containing energy drink increased the urine caffeine paraxanthine and theophylline concentrations after the game, in comparison with the pre-exercise sample (Table 1; *P*<0.05). As expected, these urinary variables remained unchanged in the control trial.

**Table 1:** Urine caffeine and paraxanthine concentrations before and after a simulated soccer game with the ingestion of a caffeinated energy drink (3 mg of caffeine/kg of body weight) or a decaffeinated control drink.

		Control	Energy Drink
Caffeine (µg • mL^−1^)	Pre	0.0±0.0	0.1±0.1
	Post	0.0±0.1	4.1±1.0* †
Paraxanthine (µg • mL^−1^)	Pre	0.6±0.6	0.6±0.7
	Post	0.3±0.3	1.5±1.0* †
Theobromine (µg • mL^−1^)	Pre	16.1±2.3	13.7±2.6
	Post	12.1±6.0	13.6±2.4
Theophylline(µg • mL^−1^)	Pre	0.1±0.1	0.1±0.1
	Post	0.1±0.1	0.4±0.1†

Data are mean ± SD for 19 soccer players.

*Different from control (*P*<0.05).

† Different from Pre (*P*<0.05).

## Discussion

The main purpose of this study was to investigate the effects of a caffeine-containing energy drink (3 mg of caffeine per kg body mass) on soccer performance. For this purpose, a commercially available sugar-free energy drink was selected to be compared with a sugar-free decaffeinated beverage. In comparison to the decaffeinated beverage, the ingestion of the caffeine-containing energy drink improved: a) mean jump height by 3.2±3.3% and power output by 3.8±4.0% during a 15-s jump test; b) mean running speed during a 7×30 m running test by 2.8±2.0%; c) total distance covered at sprint velocity by 30.0±11.3%; and d) number of sprint bouts during a simulated game from 30±10 to 24±8. Thus, the ingestion of an energy drink (in a dose of 3 mg/kg of caffeine) is a potent ergogenic aid for soccer player field performance.

Several studies have been geared to elucidate the benefits of pre-exercise caffeine ingestion on repeated sprinting ability. Carr et al. [26] investigated the effects of ingesting 6 mg/kg of caffeine on 5 sets of 6×20 m with 20–60 s of recovery between repetitions. They found that caffeine enhanced the sprint velocity in all running sets. Glaister et al. [19] also found an increased running speed with the ingestion of 5 mg/kg of caffeine on 12×30 m with 35 s of recovery between repetitions. Stuart et al. [20] reported an increase in sprinting speed in a group of rugby players that ingested 6 mg/kg of caffeine prior to a rugby specific test which included sprints. In addition, Gant et al. [27] and Roberts et al. [28] found that 4 mg/kg of caffeine augments the benefits of carbohydrates on team sports sprint performance. However, the only investigation about the effects of caffeine ingestion using energy drinks (1.3 mg/kg [14]) did not find any benefits during 3×8 “*all out*” sprints.

The current study presents some novel factors with respect to previous investigations on repeated sprint ability. First, the caffeine was provided in the form of a sugar-free energy drink and the caffeine dose investigated (3 mg/kg) was in between the doses that have been found to be ergogenic and the ones which had no benefit on performance. Second, in addition to a 7×30 m repeated sprint test, sprinting performance during a simulated soccer game was measured using GPS technology. In comparison to a decaffeinated control drink, caffeine ingestion was found to increase mean running speed during a team-sports specific test (Figure 2; *P*<0.05), running distance covered at sprint speed (Figure 4, zone 6 = 489±172 *v* 376±122 m; *P*<0.05) and the number of sprint actions during a simulated soccer game (30±10 *v* 24±8; *P*<0.05). Furthermore, the caffeinated energy drink increased the mean jump height by 1.1±0.9 cm (*P*<0.05) and the muscle power generated during a 15-s jump test by 2.3±4.6 kW (*P*<0.05), which may represent a meaningful improvement for headers or when players are competing for a ball. These data suggest the need of ingesting at least 3 mg/kg of caffeine in the form of an energy drink to increase soccer performance [15].

In this study, the average distance covered per minute of play ranged from 92±11 m · min^−1^ with the placebo drink to 98±11 m · min^−1^ with the energy drink condition. There was some inter-individual variability derived from the position of each player on the field (midfielders and forwards covered more distance than defenders) which produced medium size of the effects in the measured variables. These running paces are consistent with the ones found in soccer players recorded with GPS technology [29] but are inferior in comparison to the ones obtained with video-recording methodology (118, 105 and 109 meters) at under-15, under-17 and under-20 levels, respectively; [30]. Out of the total meters covered during a soccer game, the distance completed at sprint velocity seems the most important fitness requirement for soccer players. Mujika and co-workers [31] reported that high-intensity running for prolonged periods of time represents a discriminative variable of success in soccer performance in both young and senior players. Since the ability to sprint, recover and sprint again is a fundamental physical variable for soccer players [32], the increase in distance covered at zones 5 and 6 during a simulated game produced by the energy drink ingestion (1205±289 *v* 1436±326 m; *P*<0.05) may represent a fundamental advantage for soccer players.

The effects of caffeine on exercise performance have been typically presented as a group mean. However, a few studies have reported caffeine effects individually during short-term activities [33], [34], [35], [36]. In these studies, most but not all individuals presented an increased performance after the ingestion of caffeine (2 to 5 mg/kg). However, there were subjects with minimal ergogenic or slight ergolytic effects derived from caffeine ingestion that were categorized as “non-caffeine responders”. The physiological causes for a lack of ergogenic response to caffeine ingestion have not yet been identified. While these studies tested caffeine intake in only one performance test, in the present investigation our participants undertook three different performance tests (jump test, repeated sprint test and simulated soccer performance) which could help to better identify non-caffeine responders. We found that 17 out of 19 participants (89% of the sample) increased their performance in two or more performance tests after the ingestion of the caffeinated energy drink, so they can be classified as caffeine responders. In contrast, two subjects had a better performance in two performance tests with the decaffeinated control drink, while no subject increased performance with the decaffeinated control drink in all three performance tests. Thus, there were two participants that could be classified as non-caffeine responders but they still increased their performance after caffeine intake in one performance test. Further research is necessary to determine if the absence of ergogenicity after caffeine intake is a biological response or is the result of low re-tests reliability.

The pre-exercise meal timing and the co-ingestion of carbohydrates may have the potential to influence the effects of caffeine intake on performance. Most studies have found ergogenic effects of caffeine ingestion when this substance was ingested several hours after a pre-exercise meal [17], [18], [19], [20], [37]. Nevertheless, Skinner et al. [33] did not find an improvement with 2, 4 or 6 mg of caffeine per kg of body mass during a 2000-m rowing test when participants ingested a meal immediately before the ingestion of caffeine. These authors argued that the ingestion of the pre-exercise meal may interfere with the absorption of caffeine and thus, with potential benefits derived from this substance. In the present study, subjects had a pre-exercise meal at least 2 hours before caffeine intake to avoid the effects of this meal on caffeine absorption. On the other hand, the co-ingestion of caffeine with carbohydrates increases glucose absorption [38] and exogenous carbohydrate utilization [39], increasing the benefits of carbohydrates on performance [27], [28]. However, to exclude the effects of carbohydrates co-ingestion on the variables under investigation we used sugar-free beverages in both experimental trials.

One limitation of this study was the use of an energy drink containing taurine, glucoronolactone and B- group vitamins, while these substances were not included in the control drink. With the results of this study, it is not feasible to identify the influence of these active ingredients on the benefits derived from energy drink ingestion. It has been found that taurine may increase aerobic performance [40] and muscle contractility [41], but these effects have been obtained with an administration over a period of at least one week. Similarly, increased performance has been recorded after long-term B- group vitamin administration [42], while there is no reference to improved performance after glucoronolactone supplementation. It is still probable that caffeine needs to be combined with other functional ingredients to increase performance.

From 1984 to 2004, the use of caffeine in sports was considered doping when athletes' urinary caffeine concentration exceeded 12 µg · mL^−1^ [43]. During this period, several authors demonstrated that caffeine was ergogenic even when the urine caffeine concentration was below this threshold [44], [45], [46]. These studies suggested that athletes benefited from the effects of caffeine ingestion on performance without being penalized by antidoping authorities. In the present study, similar outcomes were found when using caffeine-containing energy drinks: the ingestion of 3 mg/kg of caffeine increased several important variables for soccer performance while mean urine caffeine concentration was only 4.1±1.0 µg · mL^−1^. These data corroborate the opinion that the previous 12 µg · mL^−1^ threshold was not valid to restrain the use of caffeine as a doping substance.

In 2004, caffeine was placed on the World Anti-doping Monitoring Program to track the trends of its use and to assess its future re-inclusion in the banned list. It has been suggested that the assessment of caffeine byproducts in urine could be a better method for identifying the use of this substance in sports. In the human body, caffeine is mostly transformed into paraxanthine (80%), theobromine (11%) and theophylline (5%), while the remaining 4% of caffeine is eliminated in urine without transformation [47]. Consequently, the assessment of the urine paraxanthine concentration might be an improved method for estimating the amount of caffeine ingested, since most of this substance is eliminated in the form of paraxanthine. However, the urinary paraxanthine concentration after the ingestion of energy drinks was lower than the urine caffeine concentration (Table 1). Since the half-life for elimination of caffeine ranges from 2.5 to 10 h in humans [48], the simulated soccer game duration was not long enough to produce the transformation of caffeine into paraxanthine. Thus, urine caffeine concentration remains the better marker to assess caffeine ingestion in the sports setting, at least in sports events shorter than 2 hours.

In summary, the ingestion of 630±52 mL of a sugar-free energy drink that contained 32 mg of caffeine per 100 mL (3 mg of caffeine per kg of body weight) increased jump height, the ability to perform repeated sprints, the total running distance during a simulated game and the distance covered at high intensity. Thus, energy drinks in the correct dosage might be an effective ergogenic aid to improve physical performance in soccer or team sports with similar physical demands to soccer. In addition, the post-exercise urinary caffeine concentration was well below the former WADA threshold for caffeine doping. If the antidoping authorities decide to reintroduce caffeine in the list of banned substances, the urinary threshold to detect the abuse of this substance should be substantially reduced.
